# Severity of Clinical Mastitis and Bacterial Shedding

**DOI:** 10.3390/pathogens12091098

**Published:** 2023-08-28

**Authors:** Isabel Krebs, Yanchao Zhang, Nicole Wente, Stefanie Leimbach, Volker Krömker

**Affiliations:** 1 Department of Bioprocess Engineering, Microbiology, Faculty II, Hannover University of Applied Sciences and Arts, 30453 Hannover, Germany; isabel.krebs@tiho-hannover.de (I.K.); yanchao.zhang@hs-hannover.de (Y.Z.); nicole.wente@hs-hannover.de (N.W.); stefanie.leimbach@hs-hannover.de (S.L.); 2 Department of Veterinary and Animal Sciences, University of Copenhagen, 1870 Frederiksberg C, Denmark

**Keywords:** clinical mastitis, dairy cow, severe mastitis, bacterial shedding

## Abstract

The aim of this cross-sectional study was to investigate associated factors of the severity of clinical mastitis (CM). Milk samples of 249 cases of CM were microbiologically examined, of which 27.2% were mild, 38.5% moderate, and 34.3% severe mastitis. The samples were incubated aerobically and anaerobically to investigate the role of aerobic and anaerobic microorganisms. In addition, the pathogen shedding was quantitatively examined, and animal individual data, outside temperature and relative humidity, were collected to determine associated factors for the severity of CM. The pathogen isolated the most was *Escherichia coli* (35.2%), followed by *Streptococcus* spp. (16.4%). Non-*aureus* staphylococci (NaS) (15.4%) and other pathogens (e.g., *Staphylococcus aureus*, coryneforms) (15.4%) were the pathogens that were isolated the most for mild mastitis. Moderate mastitis was mostly caused by *E. coli* (38%). *E. coli* was also the most common pathogen in severe mastitis (50.6%), followed by *Streptococcus* spp. (16.4%), and *Klebsiella* spp. (10.3%). Obligate anaerobes (*Clostridium* spp.) were isolated in one case (0.4%) of moderate mastitis. The mortality rate (deceased or culled due to the mastitis in the following two weeks) was 34.5% for severe mastitis, 21.7% for moderate mastitis, and 4.4% for mild mastitis. The overall mortality rate of CM was 21.1%. The pathogen shedding (back logarithmized) was highest for severe mastitis (55,000 cfu/mL) and *E. coli* (91,200 cfu/mL). High pathogen shedding, low previous somatic cell count (SCC) before mastitis, high outside temperature, and high humidity were associated with severe courses of mastitis.

## 1. Introduction

CM is a costly disease in dairy cows and has a negative impact on animal welfare [1,2]. According to the definitions of the International Dairy Federation (IDF) (2011) [3], CM is classified into three severity scores: mild, moderate, and severe. Mild mastitis is characterized by abnormalities in milk, such as flakes or abnormal color. If there are additional local signs of inflammation of the udder, like swelling, redness, and pain, it is classified as moderate. In addition to the aforementioned symptoms, severe mastitis is characterized by symptoms of a systemic disease, such as abnormalities in body temperature and behavior. Schmenger and Krömker (2020) [4] showed that mild mastitis is the most commonly occurring (55.2%), followed by moderate (35.7%) and severe mastitis (9.1%). *E. coli* is a common cause of severe mastitis [5,6,7]. The severity of *E*. *coli* mastitis is mainly determined by cow factors rather than by *E. coli* pathogenicity [5,6,8]. Polymorphonuclear neutrophils (PMN) are a part of the innate immune system and mainly influence the severity and outcome of mastitis [5,9]. Animals with an immediately increasing PMN function and SCC after infection of *E. coli* developed moderate mastitis rather than severe mastitis [9]. A study on mastitis caused by *E*. *coli* found that mastitis severity and bacterial growth in the affected udder quarter increased particularly around the time of parturition and early lactation [10]. Furthermore, the occurrence of severe mastitis is positively correlated with milk production [11,12], previous treatments with corticosteroids [12], previous occurrence of mastitis, and other diseases [12,13]. However, low milk fat content is associated with severe mastitis rather than with mild and moderate mastitis [12]. Oliveira et al. (2013) [11] found no association between previous cases of mastitis and severity of the current mastitis. Green et al. (2004) [14] described a higher risk of developing CM for cows with low SCC. In addition to host factors, the pathogen species also seems to play a role in the severity of development. Severe cases are mainly found in infections with coliforms [12] and frequently result in death of the affected animals. The mortality rate for severe cases caused by *E. coli* or *K*. *pneumoniae* is approximately 35% [15]. Environmental factors also influence the severity of CM. Severe mastitis occurs more frequently in the summer months in contrast to moderate and mild mastitis [12].

The shedding rate of mastitis-causing pathogens could be an important point in pathogen transmission to other animals in the herd [16]. Fredebeul-Krein et al. (2022) [12] showed a positive correlation between the severity of CM and the shedding intensity of the pathogens. Animal-specific factors such as the SCC and the number of lactations, as well as weather influences (e.g., heat stress) and the respective bacterial species, influence pathogen shedding. High pathogen shedding was found in animals with high SCC in the first or second lactation; these animals may serve as a source of mastitis pathogens within the herd [16]. Therefore, pathogen shedding may be an important factor in the development of mastitis. Stress factors, especially heat stress, increase the pathogen shedding of some species, for example, *Streptococcus uberis* [16]. Ziesch et al. (2016) [17] showed a correlation between shedding and bacteriological cure rate after antimicrobial treatment. The higher the shedding, the lower the probability of bacteriological cure. The shedding rate also depends on the bacterial species. Staphylococci are excreted in a lower amount than streptococci [16]. Although high shedding rates were described for *E. coli* [18] and *S. uberis* [19], the shedding for *S. aureus* is usually lower [18,20].

In most studies, the shedding rate for mastitis-causing pathogens was examined semi-quantitatively and without considering the transport time from the dairy farms to the laboratory [12,21,22]. Since the number and distribution of the microorganisms can be influenced by long storage and transport, a timely (<48 h) transport and conservation of the samples to the laboratory was an important point in this study. In this way, possible growth of or damage to the microorganisms could be kept to a minimum and a systematic influence of storage and transport on the number of microorganisms could be prevented. The aim of the present study was to investigate the association between bacterial shedding and the severity of CM and to determine an exact quantitative number of pathogens in milk at the time of the occurrence of CM. Routine diagnostics in mastitis include only the examination of aerobic microorganisms. Little is known about the role of anaerobic bacteria in CM of dairy cows. In a study about anaerobes in bovine mastitis, anaerobes were isolated in 12% of lactating cows [23]. Additionally, within the present study, the role of anaerobic microorganisms in CM of dairy cows was investigated.

## 2. Materials and Methods

All applicable guidelines for the care and use of animals were followed. An application for a license for animal testing was not required by the local government due to the study design. The study complied with the International Guiding Principles for Biomedical Research Involving Animals (1985).

### 2.1. Characteristics of Herds

This cross-sectional study was carried out in the period from July 2021 to August 2022. Milk samples from cows with CM were collected from 13 dairy farms in Lower Saxony, Germany. All of the study farms participated in the dairy herd improvement (DHI) program. The herd size ranged from 170 to 2500 Holstein Frisian cows, with an average 305 d milk yield of 11,500 kg. The average SCC of the bulk tank milk was between 150,000 and 250,000 cells/mL. The cows of all herds received total or partial mixed rations and were milked two or three times a day in a side-by-side parlor, herringbone parlor, or rotary milking parlor, or by a milking robot. The animals were kept in free-stall barns with cubicles or deep straw bedding or compost barns.

### 2.2. Sampling

The dairy farm personnel received training from the veterinarian for recognizing CM and performing correct sample collection in accordance with the IDF (2011) [3] Standard and the guidelines of the Society of Veterinary Medicine (GVA) (2018) [24]. During the milking process, farm personnel detected CM, classified its severity in accordance with the IDF guidelines, and immediately collected milk samples from the affected quarter before antimicrobial treatment in accordance with the guidelines of GVA. Samples of mild and moderate mastitis were only collected when samples from severe mastitis cases also accumulated. Before sampling, the apex of each teat was cleaned and disinfected with 70% ethanol. The samples were taken after discarding the first three streams of milk. Approximately 10 mL of milk were collected in sample tubes containing boric acid (Ly20) as a preserving agent [25]. During sampling, disposable gloves were used. Milk samples were stored at 6 °C until they were transported to the microbiological laboratory of Hannover University of Applied Sciences and Arts (Hannover, Germany) < 48 h for analysis.

### 2.3. Laboratory Procedures

Milk samples were examined in accordance with GVA (2018) [24] guidelines, which are similar to National Mastitis Council (NMC) procedures (1999) [26].

A total of 100 µL of each sample was plated in duplicate using serial dilution (10^−1^ bis 10^−4^) on esculin blood agar (Oxoid Deutschland GmbH, Wesel, Germany). Only the agar plates with 10 to 300 colonies were considered for the calculation of colony forming units per mL (cfu/mL). This resulted in a lower detection limit of 100 cfu/mL (colony forming units per milliliter). Due to the highest number of countable colonies being 300 colonies per plate, the upper detection limit was 3 × 10^6^ cfu/mL. One set was incubated aerobically and the other anaerobically at 37 °C. The aerobically incubated plates were analyzed after 24 h and 48 h. Plates to be examined for obligate anaerobes were incubated for 7 d. The grown colonies were counted quantitatively for each plate and pathogen at different dilution levels to determine an exact number of pathogens. Subsequently, the total pathogen shedding (cfu/mL) was calculated for each pathogen using the number of colonies.

The grown colonies were differentiated by means of Gram staining, morphology and cell morphology, hemolysis patterns, and esculin hydrolysis. Gram-positive catalase-positive cocci (3% H_2_O_2_, Merck KGaA, Darmstadt, Germany) were identified as staphylococci. A clumping factor test (DiaMondiaL Staph Plus Kit, Sekisui Virotech GmbH, Russelsheim, Germany) was used in β-hemolyzing staphylococci to discriminate between *S. aureus* and NaS. Catalase-negative Gram-positive esculin-hydrolyzing cocci were cultured on modified Rambach agar medium to differentiate *Enterococcus* spp. from *S. uberis*. Esculin non-hydrolyzing streptococci were classified according to the Lancefield serotyping by using the Strep Latex Kit (DiaMondiaL, Vienna, Austria).

Catalase-negative Gram-positive irregular rods with a Y-shaped cell configuration and β-hemolysis were identified as *Trueperella pyogenes*. Gram-positive, non-hemolytic catalase-positive irregular rods were defined as coryneforms. Yeasts and *Prototheca* spp. were determined by microscopy.

Gram-negative rods were differentiated by their ability to catabolize glucose under aerobic and anaerobic conditions (glucose-supplemented oxidation–fermentation test medium, Merck KGaA) and cytochrome C oxidase production (Bactident Oxidase, Merck KGaA). Cytochrome C oxidase-negative colonies fermenting glucose were cultured on Chromocult^®^Coliform Agar (Merck KGaA) to distinguish *E. coli* and other coliforms. Non-motile coliforms were determined as *Klebsiella* spp. Gram-negative, cytochrome C oxidase-positive bacteria, which metabolized glucose oxidatively, were identified as *Pseudomonas* spp.

Samples were considered to be contaminated if more than two different pathogen species were detected. The somatic cell count of the milk samples was determined by flow cytometry (SomatoScope^TM^ Smart, PerkinElmer LAS (Germany) GmbH, Rodgau, Germany).

### 2.4. Data Collection

Several risk factors associated with the severity of CM were collected for each mastitis case. Cow-associated data were collected from the herd management program and DHI records. Weather data were collected from the nearest weather station to the farm. Table 1 shows possible risk factors for the severity of CM and their categorization.

### 2.5. Statistical Analysis

The data were processed and statistically evaluated using the programs Microsoft Excel 2021 (Microsoft Corp., Redmond, WA, USA) and SPSS 28.0 Inc. (Chicago, IL, USA). Udder quarter with a clinical mastitis case was the statistical unit. Associations between the severity of occurring mastitis cases and potential associated factors (independent variables) were examined with generalized linear mixed models with logit link and binomial response (y/n (logistic regression)) after pre-screening for variable selection in univariable analysis.

The relation between dependent and independent variables was tested first by appropriate univariable tests. Multicollinearity was checked with Spearman/Kendall’s tau, which indicated a correlation of r > 0.70. For this reason, no variables were excluded. Then, independent variables associated with the dependent variable at *p* < 0.10 in the univariable test were submitted to generalized linear mixed models. Using logistic regression procedures, the association between severity and risk factors (independent variables) was examined. Herd was considered as a random effect.

A backward stepwise procedure was used to select the final multivariable regression model. Potential risk factors were excluded if *p* > 0.05.

Meaningful biological interactions between the fixed effects were also used in the final model if significant (*p* < 0.05) and if they did not increase the Akaike Information Criterion (AIC). Non-significant effects or interactions that increased the AIC were not included in the final model. Model fit was evaluated by checking the normality of the residuals.

Scaled identity was chosen as the covariance structure because it was assumed that there were no correlations between the elements. Odds ratios (OR) were calculated to describe the direction of the relationship between dependent and independent variables. ORs were determined with 95% confidence intervals (CI 95%) and statistical significance was set at *p* ≤ 0.05. The calculated number of pathogens was logarithmized (log10 cfu/mL) to obtain a normal distribution.

## 3. Results

### 3.1. Microbiological Findings

Milk samples from 249 cases of CM were examined. Milk samples from a total of 239 cases were included, of which 65 cases (27.2%) were mild, 92 (38.5%) moderate, and 82 (34.3%) severe mastitis (Table 2). In 10 cases, more than two different pathogens were detected, which were defined as contaminated and excluded from the study. Milk samples of 50 cows showed mixed infections and 5 cows had two mastitis quarters.

The most frequently isolated pathogen was *E. coli* (35.2%, 86/244), followed by *Streptococcus* spp. (16.4%, 40/244) and other pathogens (10.7%, 26/244), for example, *S. aureus* (0.8%, 2/244), *T. pyogenes*, *Proteus* spp., and coryneforms. Of all cases in which streptococci were involved, *S. uberis* was isolated in 82.5% (33/40). However, there was a high number of moderate and severe cases of mastitis compared to mild mastitis. *Streptococcus* spp. (15.4%, 10/65), NaS (15.4%, 10/65), and other pathogens (15.4%, 10/65) were the most isolated pathogens for mild mastitis. Nonetheless, mixed infections mostly occurred in mild mastitis, in which mainly NaS and other pathogens were involved. Moderate mastitis was mostly caused by *E. coli* (38%, 35/92). *Streptococcus* spp. (17.4%, 16/92) and *Klebsiella* spp. (14.1%, 13/92) were also frequently involved in moderate mastitis. *E. coli* was also the most common pathogen in severe mastitis (50.6%, 44/87), followed by *Streptococcus* spp. (16.1%, 14/87) and *Klebsiella* spp. (10.3%, 9/87). The pathogen distribution in anaerobically incubated milk inoculum was similar to that of the aerobe examination. Obligate anaerobes (*Clostridium* spp.) were isolated in one case (0.4%, 1/247) of moderate mastitis.

### 3.2. Descriptive Results of Variables

The pathogen shedding increased with the severity of CM. The lowest shedding rate was detected for mild mastitis (1500 cfu/mL, back logarithmized). Severe mastitis showed the highest shedding rates of 55,000 cfu/mL (Table 3). The upper limit of detection for the pathogen shedding (3 × 10^6^ cfu/mL) was reached in at least one of the 238 cases of CM for all pathogen species except for NaS. The highest average pathogen shedding was detected for *E. coli* (91,200 cfu/mL), followed by *Klebsiella* spp. (70,800 cfu/mL) and *Streptococcus* spp. (58,900 cfu/mL). The lowest shedding rates were shown by other pathogens (3500 cfu/mL) and NaS (1100 cfu/mL). 

Severe mastitis showed the highest shedding rates for each pathogen species. In severe and mild mastitis, the highest shedding rates were found for *E. coli*. In moderate mastitis, the highest shedding rate was determined for *Klebsiella* spp. The lowest shedding rate was detected for NaS in every severity score. Overall, the shedding rate for *E. coli*, *Streptococcus* spp., and *Klebsiella* spp. increased with increasing severity. Table 3 shows the pathogen shedding in relation to the pathogen species and severity of CM.

Individual SCC were collected for 214 cases from the DHI records of the last three months before the onset of mastitis. The occurrence of severe and moderate mastitis decreased with increasing previous SCC. The likelihood of developing severe mastitis increased with low SCC before the onset of the disease. Most cases with low SCC (≤100,000 cells/mL) before mastitis developed severe (48.4%, 46/95) and moderate mastitis (32.6%, 31/95) and 18.9% showed mild mastitis. A total of 56.5% (13/23) of cases with high SCC (>1,000,000 cells/mL) before the disease were mild mastitis.

**Figure 1:**
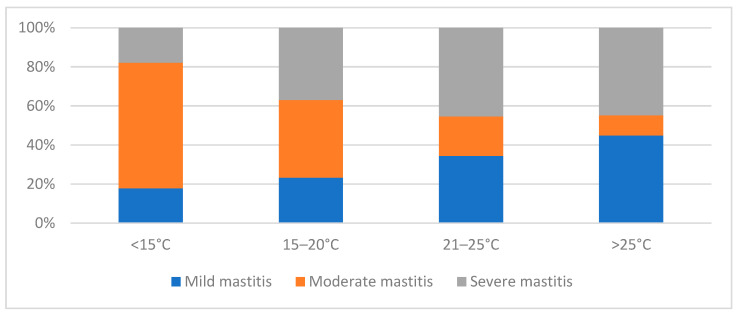
Severity of clinical mastitis in relation to the outside temperature.

The percentage of mild and severe mastitis increased with increasing outside temperatures (Figure 1). Of all cases of mastitis that occurred at temperatures of over 25 °C, most were severe (44.8%, 13/29) and mild (44.8%, 13/29) mastitis. The occurrence of moderate mastitis decreased with increasing temperature. Moderate mastitis was most common at temperatures below 15 °C (64.4%, 47/73).

**Figure 2:**
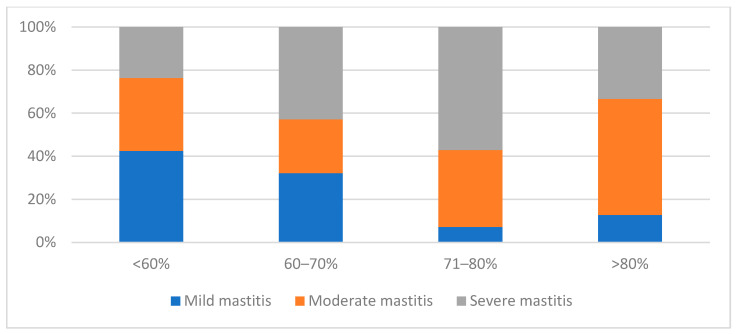
Severity of clinical mastitis in relation to the relative humidity.

In general, the proportion of moderate and severe mastitis increased with increasing relative humidity (Figure 2). At high humidity, the likelihood of developing moderate and severe mastitis increased. At humidity over 80%, moderate mastitis occurred most frequently (54%, 34/63). The proportion of severe mastitis was highest at humidity between 71% and 80% (57.1%, 24/42) and lowest at humidity below 60% (23.6%, 25/106). The occurrence of mild courses decreased with increasing humidity. Mild mastitis was most common at low humidity of less than 60% (42.5%, 45/106).

Follow-up data for the outcome of the CM were collected for 123 cows for the following two weeks after the disease occurred. In 66.7% (82/123) of cases, the affected animals survived the mastitis; 12.2% (15/123) died or left the farm due to other reasons (e.g., claw diseases, low milk yield). A total of 21.1% (26/123) of the animals died (deceased or culled) due to the mastitis, of which 73.1% (19/26) was severe mastitis. The mortality rate increased with increasing severity of clinical mastitis. The mortality rate was 34.5% (19/55) for severe mastitis, 21.7% (5/23) for moderate mastitis, and 4.4% (2/45) for mild mastitis.

Most CM cases within this study were obtained from dairy cows, which were at least in their second lactation (88.3%, 211/239) and their first third of lactation (37.2%, 89/239). All in all, 51.5% (101/196) of cases showed an average milk yield of 10,000 to 12,000 kg per lactation and a daily milk yield of 30 to 40 kg (42.7%, 97/227) and over 40 kg (42.7%, 97/227). Most cases showed in the last DHI report a milk protein content of 3 to 3.5% (57.2%, 119/208) and a milk fat content of 3 to 4% (59.1%, 123/208). Most affected animals with CM had no previous illnesses (62.9%, 149/237) and had not received any previous treatments in the previous 30 days (83.4%, 206/247).

### 3.3. Results of the Multivariable Statistic

A generalized linear mixed model was performed to analyze the associations between risk factors and severity of CM after pre-screening for variable selection in univariable analysis (Table 4). The likelihood of the occurrence of severe mastitis increased with high shedding rates. High shedding rates (<100,000 cfu/mL) showed higher odds for developing severe mastitis (OR = 2.797, CI 1.029–7.607) than low shedding rates. Animals with low SCC (≤100,000 cells/mL) before mastitis occurred (OR = 3.614, CI 1.703–7.673) showed a higher probability of developing severe mastitis than animals with high SCC. High outside temperature and humidity promoted the occurrence of severe mastitis. Odds for temperatures of >25 °C (OR = 4.877, CI 1.289–18.460) and humidity of >80% (OR = 3.217, CI 0.761–13.607) were higher than for low values. The statistical analysis showed no association between temperature–humidity index (THI) and severity of CM.

## 4. Discussion

The study showed a severity distribution of 27.2% mild, 38.5% moderate, and 34.3% severe mastitis. Other studies detected an opposite distribution, with a higher prevalence of mild mastitis than severe cases [4,11]. One reason for these different results could be unrecorded cases on dairy farms. It is possible that the farms did not recognize every mild mastitis case. Furthermore, samples of mild and moderate mastitis were only collected when samples of severe cases also accumulated.

The severity of CM showed an association with the pathogen species. NaS and other pathogens were the most frequently isolated pathogens in mild mastitis, coliforms, and streptococci in moderate and severe mastitis. In the literature, *Streptococcus* spp., followed by *E. coli*, were the most common causes of severe mastitis [4,7,12]. The results also depend on the dairy herds, because each farm has different microbial flora. Furthermore, moderate and severe cases of CM were recorded the most in this study, which also influences the distribution of isolated pathogens. In general, streptococci and *E. coli* are particularly common causes of severe mastitis [11,12,13]. However, it is known that mild mastitis is mostly caused by minor pathogens such as NaS [28]. Nevertheless, in this study, mild cases were also caused by *E. coli* but with a generally lower shedding rate. Studies focusing on the pathogenicity and virulence factors of *E. coli* showed no association with the severity of CM [5]. The PMN function of the animal’s immune system is crucial to the outcome of CM [9]. Host factors such as stage of lactation and parity seem to influence the severity of CM more than the pathogenicity of *E. coli* [5]. Dairy cows with an immediately increasing PMN function after an infection with *E. coli* prevent rapid bacterial growth, which could explain the low shedding rate in these mild cases. On the dairy farms participating in this study, obligate anaerobes seem to be of little importance for CM.

In this study, a positive association between shedding rate and severity of CM was determined, which agrees with the results of Fredebeul-Krein et al. (2022) [12]. Severe mastitis showed the highest shedding rates and mainly occurred at high outside temperatures and humidity values. Dairy cows that suffer from heat stress show higher shedding rates [16]. High outside temperatures and humidity may increase the growth of pathogens in the environment (e.g., in the litter), which could result in a higher infection pressure and pathogen colonization in the udder. In severe cases, there is a risk of bacteremia and subsequent sepsis, which can occur if bacteria enter the bloodstream [7,15,29]. It is known that pathogens can open the blood–milk barrier (BMB) [30,31]. In mastitis cases with high shedding rates, many pathogens are present in the udder, which could suggest a high probability of a destroyed BMB. Another explanation could be a stronger immune response to an increasing bacteria population in the udder, which results in severe mastitis. The question remains why the bacteria multiply so rapidly in some mastitis cases. One reason could be a diverging PMN function in dairy cows. Dairy cows with an immediately increasing PMN function and SCC after an intramammary infection develop moderate mastitis rather than severe mastitis [9]. A slower immune response of the PMN could result in a stronger multiplication of pathogens, which could also explain the higher shedding rates in severe cases. A strong immune response is required to destroy the pathogens, which could explain the severe signs of inflammation in severe mastitis. The pathogen species also influenced the shedding rate. *E. coli* showed the highest shedding rates, followed by *Klebsiella* spp. and *Streptococcus* spp. In the literature, coliforms and streptococci show the highest shedding rates and NaS and S. *aureus* the lowest shedding rates [12,16]. In many cases of CM, the upper detection limit of the pathogen shedding was reached. Therefore, the shedding rate seems to be higher than previously assumed. In further studies, higher dilution levels should be considered to determine shedding rates for cases with particularly high bacterial counts.

The study showed that affected cows with low SCC (≤100,000 cells/mL) before the onset of the mastitis were more susceptible to developing severe mastitis than cows with high SCC (>100,000 cells/mL). The risk of developing severe mastitis compared with mild mastitis decreased for cows with high individual SCC in the month before CM was diagnosed [32]. Green et al. (2004) [14] described a higher risk of developing CM for cows with low SCC. These results were also confirmed by studies that examined bulk milk SCC in relation to the incidence of CM in a herd [33,34]. One possible explanation could be a slower immune response to the pathogens entering the udder for dairy cows with a previously low SCC. A slower immune response and PMN function could facilitate the proliferation of pathogens in the udder. Therefore, high SCC could prevent an excessive multiplication of pathogens. The SCC is a parameter for inflammation of the udder tissue and mainly varies due to fluctuations in the number of leukocytes, which shows a direct response to present pathogens. Few studies described a protective effect of high SCC and previous infections with minor pathogens (e.g., *Corynebacterium bovis*) on new intramammary infections [35]. Another explanation could be that subclinical mastitis is characterized by high SCC and may temporarily develop to CM. The chronicity of subclinical mastitis could be an associated factor for the severity of CM and pathogen shedding. Further studies are required to investigate this assumption.

The severity of CM was significantly associated with the outside temperature and humidity. The higher the temperature and humidity, the higher the probability of developing severe mastitis. In a study by Hamel et al. (2021) [16], the effect of heat stress on mastitis in dairy cows was investigated. The THI was calculated as a representative factor for heat stress. The study showed a negative impact of heat stress on udder health and increasing pathogen shedding for high THI levels (>60). In humid climates, the relative humidity is the limiting factor of heat stress, and the animals suffer from heat stress, especially when relative humidity is high [27]. Cows use transpiration for thermoregulation. At high temperatures with high humidity, it is more difficult to regulate body temperature than at low humidity. Therefore, high relative humidity amplifies the effect of outside temperature on heat stress. The latter could have a negative impact on the immune system. Elvinger et al. (1992) [36] showed a decreased migration of leukocytes to the mammary gland due to heat stress.

The mortality rate increased with increasing severity of clinical mastitis. Severe mastitis showed the highest mortality rate. One possible reason is the risk of developing bacteremia or sepsis in severe mastitis [7,15,29], which is also associated with a high mortality rate [15,29]. Furthermore, a negative impact of infections with *E. coli* and *K. pneumoniae* on survival was described [15,29], and in the present study, *E. coli* was the most frequently isolated pathogen in cases of severe mastitis.

## 5. Conclusions

*E. coli* was isolated the most in milk samples of cases of severe mastitis, followed by *Streptococcus* spp. And *Klebsiella* spp. Obligate anaerobes seem to be of little importance for CM. Severe mastitis showed the highest pathogen shedding (55,000 cfu/mL) and was promoted by previously low SCC, high outside temperatures, and high relative humidity. The shedding intensity was highest for *E. coli* (91,200 cfu/mL) and *Klebsiella* spp. (70,800 cfu/mL). The mortality rate was higher for severe mastitis (34.5%) than for moderate (21.7%) and mild mastitis (4.4%). Based on these results, the prevention of severe mastitis could be improved.

## Figures and Tables

**Table 1:** Potential risk factors of clinical mastitis at the animal and environment levels.

Risk Factor	Description	Categories
Number of lactations	Lactation number in which mastitis appeared	1 and >1
Days in milk	Lactation trimester in which mastitis appeared	1. Third (<100 d) 2. Third (100–200 d) 3. Third (>200 d)
Average SCC ^1^ before mastitis occurred	Average SCC calculated from SCCs of the last 3 months	≤100,000 cells/mL >100,000 cells/mL
Average 305 d milk yield	Average 305 d milk yield calculated from all lactations. Only dairy cows with completed lactations were included.	<10,000 kg 10,000–12,000 kg >12,000 kg
Daily average milk yield	Daily average milk yield in the current lactation	<30 kg 30–40 kg >40 kg
Protein content	Last protein content of the milk	<3% 3–3.5% >3.5%
Fat content	Last fat content of the milk	<3% 3–4% >4%
Previous illnesses	Previous diseases of the affected dairy cow in current lactation	None, mastitis, claw disease, fertility disease
Pretreatments	Treatments in the last 30 days with indication of the active substance	None, antibiotics, NSAID ^2^
Follow-up data for the outcome of the clinical mastitis	Data of the affected cow for the following two weeks after the day of mastitis detection	Survived, died (deceased, euthanized, culled due to this mastitis), other (culled or left due to reasons other than current mastitis case)
Outside temperature	Outside temperature measured on the day of mastitis at 12 o’clock	<15 °C 15–20 °C 21–25 °C >25 °C
Relative humidity	Humidity measured on the day of mastitis at 12 o’clock	<60% 60–70% 71–80% >80%
Temperature–humidity index (THI) ^3^	Value calculated from outside temperature and humidity	No categorization

^1^ Somatic cell count. ^2^ Non-steroidal anti-inflammatory drug. ^3^ THI was calculated based on the following equation (Bohmanova et al., 2007) [27]: THI = (1.8 × T + 32) − (0.55 − 0.0055 × RH) × (1.8 × T − 26) (T = outside temperature (°C), RH = relative humidity (%)).

**Table 2:** Isolated pathogens of aerobically incubated milk samples and number of clinical mastitis cases in relation to the severity of mastitis.

Severity (n)	*E. coli* ^1^	*Streptococcus* spp.	*Klebsiella* spp.	NaS ^2^	Other	Mixed Infections	No Growth
Mild (65)	7 (10.8%)	10 (15.4%)	3 (4.6%)	10 (15.4%)	10 (15.4%)	25 (38.4%)	0
Moderate (92)	35 (38.1%)	16 (17.4%)	13 (14.1%)	0	12 (13%)	13 (14.1%)	3 (3.3%)
Severe (87 ^3^)	44 (50.6%)	14 (16.1%)	9 (10.3%)	1 (1.2%)	4 (4.6%)	12 (13.8%)	3 (3.4%)
Total (244 ^3^)	86 (35.2%)	40 (16.4%)	25 (10.2%)	11 (4.5%)	26 (10.7%)	50 (20.5%)	6 (2.5%)

^1^ *Escherichia coli*. ^2^ Non-*aureus* staphylococci. ^3^ Five cases with two quarters of mastitis.

**Table 3:** Average pathogen shedding in relation to the severity of clinical mastitis and pathogen species.

Average Pathogen Shedding (cfu/mL) ^1^						
Severity	*E. coli* ^2^	*Streptococcus* spp.	*Klebsiella* spp.	NaS ^3^	Other	Total
Mild	1400 (r ^4^ = 100–3,000,000; sd ^5^ = 827,322)	4400 (r = 185–3,000,000; sd = 723,658)	3200 (r = 100–167619; sd = 68,843)	1000 (r = 100–900,000; sd = 181,342)	1000 (r = 100–61904; sd = 11,155)	1500 (r = 100–3000000; sd = 449,888)
Moderate	89,100 (r = 100–3,000,000; sd = 1,249,439)	51,300 (r = 100–3,000,000; sd = 1,344,815)	72,400 (r = 100–3,000,000; sd = 1,395,108)	100 (r = 100; sd = 0)	21,900 (r = 100–3,000,000; sd = 1,172,014)	21,000 (r = 100–3,000,000; sd = 1,241,577)
Severe	269,200 (r = 100–3,000,000; sd = 1,480,277)	457,000 (r = 150–3,000,000; sd = 1,320,246)	229,100 (r = 270–3,000,000; sd = 1,426,254)	2000 (r = 100–535,000; sd = 231,163)	3500 (r = 100–1,855,000; sd = 741,182)	55,000 (r = 100–3,000,000; sd = 1,425,957)
Total	91,200 (r = 100–3,000,000; sd = 1,281,073)	58,900 (r = 100–3,000,000; sd = 1,370,237)	70,800 (r = 100–3,000,000; sd = 1,413,249)	1100 (r = 100–900,000; sd = 188,612)	3500 r = 100–3,000,000; sd = (867,637)	

^1^ Back logarithmized. ^2^ *Escherichia coli*. ^3^ Non-*aureus* staphylococci. ^4^ Minimum–maximum. ^5^ Standard deviation.

**Table 4:** Generalized linear mixed model for the probability of developing severe mastitis in dependence on the variables.

Risk Factor	Categories	Regression Coefficient	Standard Error	t-Value	*p*-Value	OR ^1^	95% CI ^2^
Intercept		0.224	0.8295	0.270	0.787	1.251	0.244–6.415
Pathogen shedding (cfu/mL)	<100,000	−0.972	0.4729	−2.056	0.041	0.378	0.149–0.960
	100,000–3,000,000	Reference					
	>3,000,000	1.029	0.5078	2.026	0.044	2.797	1.029–7.607
Somatic cell counts before mastitis (cells/mL)	≤100,000	1.285	0.3821	3.363	<0.001	3.614	1.703–7.673
	>100,000	Reference					
Outside temperature (°C)	<15	−3.112	0.6711	−4.637	<0.001	0.045	0.012–0.167
	15–20	Reference					
	21–25	1.412	0.5137	2.748	0.006	4.103	1.491–11.289
	>25	1.585	0.6756	2.346	0.020	4.877	1.289–18.460
Relative humidity (%)	<60	−2.238	0.6910	−3.238	0.001	0.107	0.027–0.416
	60–70	Reference					
	71–80	0.146	0.7390	0.198	0.844	1.157	0.270–4.963
	>80	1.168	0.7320	1.596	0.112	3.217	0.761–13.607

^1^ Odds ratio. ^2^ 95% confidence interval for odds ratio.

## Data Availability

The data presented in this study are available upon request from the corresponding author. The data are not publicly available due to privacy.
